# *Peloplasma aerotolerans* gen. nov., sp. nov., a Novel Anaerobic Free-Living Mollicute Isolated from a Terrestrial Mud Volcano

**DOI:** 10.3390/life14050563

**Published:** 2024-04-26

**Authors:** Maria A. Khomyakova, Alexander Y. Merkel, Andrei A. Novikov, Alexander I. Slobodkin

**Affiliations:** 1 Winogradsky Institute of Microbiology, Research Center of Biotechnology of the Russian Academy of Sciences, Leninskiy Prospect, 33, bld. 2, 119071 Moscow, Russia; 2 Department of Physical and Colloid Chemistry, Gubkin University, Leninskiy Prospect, 65/1, 119991 Moscow, Russia

**Keywords:** free-living mollicutes, anaerobe, aerotolerance, cell wall-less bacteria, *Acholeplasmataceae*

## Abstract

A novel aerotolerant anaerobic bacterium (strain M4Ah^T^) was isolated from a terrestrial mud volcano (Taman Peninsula, Russia). Cells were small, cell-wall-less, non-motile cocci, 0.32–0.65 μm in diameter. The isolate was a mesophilic, neutrophilic chemoorganoheterotroph, growing on carbohydrates (D-glucose, D-trehalose, D-ribose, D-mannose, D-xylose, D-maltose, D-lactose, D-cellobiose, D-galactose, D-fructose, and D-sucrose), proteinaceous compounds (yeast extract, tryptone), and pyruvate. Strain M4Ah^T^ tolerated 2% oxygen in the gas phase, was catalase-positive, and showed sustainable growth under microaerobic conditions. The dominant cellular fatty acids of strain M4Ah^T^ were C_16:0_ and C_18:0_. The G+C content of the genomic DNA was 32.42%. The closest phylogenetic relative of strain M4Ah^T^ was *Mariniplasma anaerobium* from the family *Acholeplasmataceae* (order *Acholeplasmatales*, class *Mollicutes*). Based on the polyphasic characterization of the isolate, strain M4Ah^T^ is considered to represent a novel species of a new genus, for which the name *Peloplasma aerotolerans* gen. nov., sp. nov. is proposed. The type strain of *Peloplasma aerotolerans* is M4Ah^T^ (=DSM 112561^T^ = VKM B-3485^T^ = UQM 41475^T^). This is the first representative of the order *Acholeplasmatales*, isolated from a mud volcano.

## 1. Introduction

*Mollicutes* is a class of bacteria characterized by the absence of a cell wall and small genome sizes (ranging from 1.2 Mb to 1.89 Mb according to Genbank database). The lack of cell walls makes them unsusceptible to some antibiotics, and is responsible for their pleomorphic form. Small genome sizes and limited biosynthetic capabilities explain the parasitic or saprotrophic existence of these microorganisms. One of the orders in this class, *Acholeplasmatales*, consists of the sole family *Acholeplasmataceae*, which mainly includes facultatively anaerobic commensals of vertebrates, insects, plants, or animals [1]. A rare free-living representative of this family—*Mariniplasma anaerobium*—was isolated from anoxic bottom water of a brackish meromictic lake and demonstrated an obligately anaerobic lifestyle [2]. The broad classification of microorganisms as anaerobic or aerobic is based on the types of reactions they employ to generate energy for growth. The majority of obligate anaerobes cannot grow in the presence of oxygen since O_2_ is toxic for them. However, some anaerobic bacteria can protect themselves from reactive oxygen species using different biochemical mechanisms [3]. Such anaerobes are considered as aerotolerant and differ considerably in their sensitivity to oxygen. Some aerotolerant anaerobes can proliferate at 10% and even higher O_2_ levels. Aerotolerant anaerobic species are found among various physiological and phylogenetic groups of prokaryotes, e.g., acetogenic bacteria, lactic acid bacteria, *Spirochaetota*, *Bacteroidota*, sulfate-reducing bacteria, and methanogenic archaea [4].

Mud volcanism is a geological phenomenon that plays a significant role in the atmospheric budget of methane [5]. Mud volcanoes are widespread throughout the globe both offshore and onshore. Thus, terrestrial mud volcanoes (TMVs) can provide a direct way to recover subsurface microbial communities due to the emission of mud, breccias, liquids, and gases from deep reservoirs to the surface through fracture networks extending to a depth of several kilometers. Thus, TMVs are geologically connected with deep subsurface petroleum and natural gas reservoirs, supplying prokaryotic communities with various organic and inorganic compounds, which can be used in microbial metabolism. Studies of microbial communities in TMVs based on molecular approaches have suggested that mainly anaerobic microorganisms play the central roles in these ecosystems [6,7,8]. A number of lithoautotrophic, sulfate-, sulfur-, and Fe (III)-reducing bacteria have been isolated from TMVs [9,10]. The information on anaerobic organoheterotrophic fermentative microorganisms, isolated from TMVs, is limited to a few reports [11,12]. Representatives of the class *Mollicutes* have been detected by molecular methods based on a microbial community analysis in several TMVs [13,14]; however, to date, no cultivated *Mollicutes* from mud volcanoes are known. The Taman Peninsula is among the regions with the most pronounced mud volcanism; the Kerch–Taman mud volcano province includes over 100 active TMVs [15]. 

In this study, we isolated from TMV a free-living, cell wall-less anaerobic strain, described its phenotypic and genomic properties, and proposed to assign it to a novel genus and species within the family *Acholeplasmataceae* as *Peloplasma aerotolerans* gen. nov., sp. nov. 

## 2. Materials and Methods

### 2.1. Enrichment and Isolation

**Figure 1:**
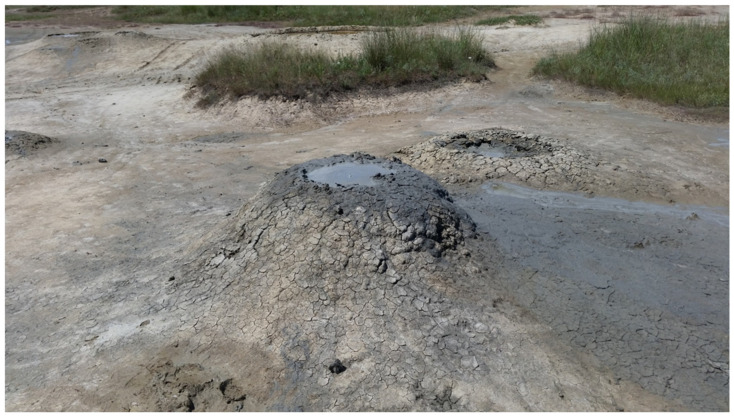
The image of the study site. A gryphon located at Gnilaya Gora mud volcano field. The diameter of the upper part of the gryphon is about 0.5 m.

Strain M4Ah was isolated from the enrichment culture containing “*Methanocrinis alkalitolerans*” as the dominant population [16]. This enrichment culture was obtained from a sample of mud collected from the active gryphon (ca. 0.7 m height, 0.5 m in diameter, Figure 1) of terrestrial mud volcano Gnilaya Gora, Taman Peninsula, Krasnodar region, Russia. Coordinates of the sampling point were 45.251° N, 37.436° E. Samples were collected in May 2017. A closed plastic 50 mL tube (Falcon, Sarstedt, Germany) was manually placed into the gryphon to a depth of 20 cm from the surface, and then the tube was opened to completely fill it with the surrounding mud. pH of the mud was 8.5, and the temperature was 21 °C. The mud contained 15.7 mM Cl^−^ and 5.3 mM SO_4_^2−^.

An enrichment culture was initiated by the 10% (*w*/*v*) inoculation of a mud sample into an anaerobic sterile medium with acetate (20 mM) as a substrate and ampicillin (0.5 g L^−1^) for the isolation of archaeal species *Methanothrix* and suppression of bacterial growth. During the isolation of *Methanothrix*-like cells for appr. 1 year, strain M4Ah was isolated as a satellite bacterium with the relative abundance of 2.5% [16]. A further enrichment and isolation of the strain M4Ah were performed in the anaerobic liquid medium of the following composition (per liter of distilled water): KH_2_PO_4_, NH_4_Cl, KCl, and MgCl_2_·6H_2_O (0.33 g each); CaCl_2_·6H_2_O, 0.033 g; NaCl, 10.00 g; NaHCO_3_, 2.00 g; and 1 mL of a trace element solution [16] and 1 mL of a vitamin solution [17] and resazurin solution (0.001 g L^−1^). The medium was boiled and cooled under N_2_ flow before adding NaHCO_3_, vitamins, and Na_2_S·9H_2_0 (0.5 g L^−1^). The medium was dispensed in 10 mL aliquots into 17 mL test tubes for anaerobic cultivation (Hungate tubes, Bellco, New Jersey, USA), equipped with butyl rubber septa, and screw caps. The head space was filled with N_2_ (high-purity grade, 100% *v*/*v*). The medium was autoclaved at 1 atm and 121 °C for 30 min. The pH of the sterile medium was adjusted to 8.0 at 25 °C with a 10% sterile anaerobic NaOH solution if necessary. A mixture of peptone, yeast extract, and glucose (1 g L^−1^ each) (PYG) from the sterile anoxic stock solution was added as the growth substrate to a final concentration of 1 g L^−1^ each before inoculation.

Cultures have been incubated in a 10 mL medium in Hungate tubes under an atmosphere of N_2_ (100%) unless otherwise mentioned. All transfers and sampling of cultures were performed with syringes and needles. Unless otherwise mentioned, the culture was incubated at 30 °C. 

### 2.2. Morphological, Chemotaxonomic, and Physiological Features

Direct counting with a phase-contrast microscope (Olympus CX-41) was used for the determination of bacterial growth. TEM was performed in the UNIQEM Collection Group (Fundamentals of Biotechnology, Moscow, Russia) using an electron microscope (JEM-100, JEOL, Tokyo, Japan). Growth experiments were performed in duplicate. The effects of temperature, pH, and salinity on growth were examined in the non-reduced medium with PYG as a growth substrate as described previously [10]. Sulfide concentration was determined colorimetrically with dimethyl-*p*-phenylenediamine [18]. The presence of catalase was estimated by the decomposition of a 3% H_2_O_2_ solution, accompanied by the foam formation when added to the biomass of the isolate.

Autoclaved anoxic stock solutions of soluble substrates were added to the sterile medium prior to culture inoculation. S^0^ was added during the preparation of the non-reduced medium and autoclaved at 0.5 atm. The medium with poorly crystalline Fe (III) oxide (ferrihydrite) was prepared as described previously [10]. Gas Chromatography equipped with a HayeSep N 80/100 mesh column at 40 °C and flow rates of 20 mL min^−1^ (with argon as a carrier gas) was used for the detection of metabolic gaseous products, produced by the strain. The ability of the strain to grow aerobically was checked in an aerobic medium containing 100% air in the gas phase. 

Sensitivity to antibiotics was tested at a 0.1 g L^−1^ concentration under optimal growth conditions.

Additionally, 10 mM MgSO_4_, S^0^ (5 g L^−1^) or 10 mM Na_2_S_2_O_3_, 10 mM antraquinone-2,6-disulfonate (AQDS), 10 mM Na_3_AsO_4_, 2.5 mM NaNO_2_ or 10 mM KNO_3_, and 10 mM ferrihydrite were tested as potential electron acceptors with PYG or pyruvate as an electron donor. Oxygen as an acceptor was tested at two different concentrations: 2% (*v*/*v*) in the gas phase of the non-reduced medium or 20% (*v*/*v*) in the gas phase of the aerobically prepared medium. 

Cellular fatty acid profiles were determined by GC-MS (Thermo Scientific Trace GC Ultra DSQ II, HP-5MS column, *E*_I_70 eV) of methyl ester derivatives prepared from 5 mg of freeze-dried cell material treated by anhydrous HCl/MeOH, based on retention time (using Supelco FAME calibration mixture, Sigma), reference equivalent chain length values [19], and mass spectra (NIST MS Search 2.0 program provided with the GC-MS setup). Cellular fatty acid profiles were determined as percentages of the total ion current peak area. Quinones were analyzed as described by Collins et al. using Finnigan 123 LCQ Advantage MAX APSI/MS [20]. Cells grown on PYG were used for the analysis. DNA G+C content was determined from the genomic data.

### 2.3. Phylogeny, Comparative Genomics, and Genome Analysis

Genomic DNA isolation and 16S rRNA gene amplification and sequencing were performed as described previously [10]. The GenBank accession number for the 16S rRNA gene sequence of strain M4Ah is OR436924. The 16S rRNA gene sequence of the isolate was compared with other sequences in GenBank [21] by using the BLAST program [22] and by means of the EzBioCloud server (http://www.ezbiocloud.net; accessed on 25 February 2020) to identify its closest relatives. The MiSeq system (Illumina, San Diego, CA, USA) with the reagent kit providing for 2 × 250 bp reading was used for sequencing of the complete genome of the strain. The RAST/SEED v2.0 pipeline [23] and NCBI’s Prokaryotic Genome Annotation Pipeline (PAGP) [24] were applied for the search of genes of interest as well as for annotation. This Whole Genome Shotgun project has been deposited in DDBJ/ENA/GenBank under the accession JASCXW000000000. For genome-based phylogenetic reconstructions, 120 bacterial single-copy marker genes (bac120) were used, as described previously [25]. For 16S rRNA gene-based phylogenetic analyses, sequences were aligned by MAFFT v7.427 (G-INS-i strategy) [26]. For bac120-based phylogenetic analyses, sequences were aligned by GTDB-Tk 2.3.2 [27]. The trees were built using the IQ-TREE program [28] with fast model selection via ModelFinder [29], the ultrafast approximation for phylogenetic bootstraps [30], and the approximate likelihood-ratio test for branches [31]. ANI values were calculated employing the EZbiocloud tool [32]; AAI values were calculated by using an online tool developed by the Kostas group at the Georgia Institute of Technology [33]. The classification of hydrogenase types and their catalytic subunits was analyzed by the HydDB program [34].

## 3. Results

### 3.1. Enrichment and Isolation

The strain M4Ah was isolated from a culture highly enriched in the methanogenic archaeon “*M. alkalitolerans*” [16]. After serial 10-fold dilutions in the same liquid medium performed consequently for three times, profiling of 16S rRNA gene amplicons by using whole-genome sequencing and a metagenomic bioinformatics analysis in the highest growth positive dilution (10^−7^) revealed the presence of sequences related to *Methanothrix* and the *Acholeplasmataceae* bacterium with the relative abundance of 97.5 and 2.5%, respectively [16]. During the cultivation of this co-culture on the PYG medium, multiple small round cells were observed in the light microscope. The co-culture was filtered using 0.45 μm pore size syringe filters (Millipore) followed by serial 10-fold dilutions in the medium containing PYG and ampicillin (0.5 g L^−1^). The culture in the last positive dilution (10^−8^) contained morphologically homogeneous non-motile small cocci without *Methanocrinis* cells and was designated as strain M4Ah. Attempts to obtain separate colonies were unsuccessful either with 1% Gelrite gellan gum or with 1.5% agar as the solidifying agent. Phase-contrast microscopy together with a molecular analysis of complete 16S rRNA gene and genome sequencing were used to confirm the purity of the strain. 

### 3.2. Phenotypic and Chemotaxonomic Characteristics

**Figure 2:**
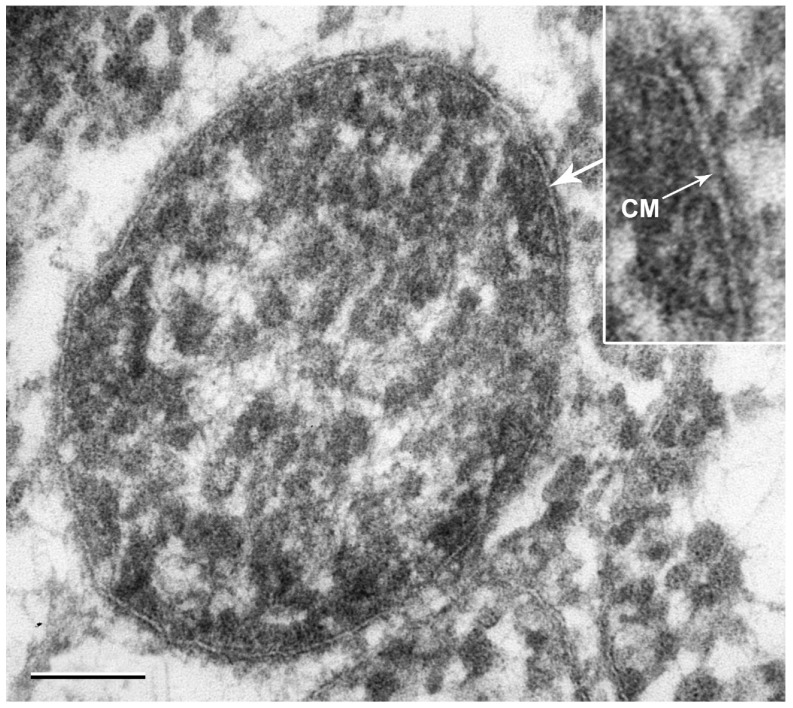
The electron micrograph of strain M4Ah, showing the absence of the cell wall. CM—cytoplasmic membrane composed of two electron-dense layers with a less dense layer in between this three-layered structure. Bar, 0.1 μm.

Cells of strain M4Ah were small irregular cocci ranging in diameter from 0.32 to 0.65 μm. Cells occurred singly and were non-motile. The cells had no cell wall, which is typical for mollicutes (Figure 2). Endospore formation was not observed. 

The temperature range for growth was 15–37 °C (optimum: 30 °C). After 30 days of incubation, we failed to determine the growth of the isolate either at 42 °C (and above) nor at 10 °C (or below). The isolate grew in the pH range from 6.5 to 10.0, and had an optimum at pH 7.0–7.5. At pH values lower than 6.0 or higher than 10.5, we were unable to detect significant growth. NaCl concentrations up to 4.0% (*w*/*v*) (with optimal NaCl concentration at 1.0%) were appropriate for strain M4Ah, while at 5.0% (*w*/*v*) NaCl and above, no growth was determined. 

The yeast extract was necessary for the growth of the strain at the concentration of at least 0.05 g L^−1^. Acetate, ethanol, and CO_2_ were produced during PYG fermentation; the doubling time under these conditions consisted of 7.9 h.

Strain M4Ah utilized the yeast extract, tryptone (pancreatic digest of caseine; Roth), pyruvate, D-glucose, D-trehalose, D-ribose, D-mannose, D-xylose, D-maltose, D-lactose, D-cellobiose, D-galactose, D-fructose, and D-sucrose (2 g L^−1^ each). No growth was observed on peptone (peptic digest of animal proteins; Fluka BioChemika), glycine, glutamate, citrate, succinate, fumarate, malate, lactate, casamino acids, beef extract, glycerol, acetate (microaerobically), D-raffinose, formate, L-arabinose, i-inositol, propionate, crotonate, and CO_2_:H_2_ under anaerobic conditions.

Strain M4Ah was able to grow under microaerobic conditions (2.0% O_2_), reaching the maximal cell concentration of 3.0 × 10^7^ cells mL^−1^. In the absence of oxygen, in the non-reduced liquid medium, the growth of strain M4Ah was two times lower (1.5 × 10^7^ cells mL^−1^). However, oxygen consumption was insignificant and constituted only 0.2%. Aerobic growth at the atmospheric concentration of oxygen was not observed. The strain M4Ah was catalase-positive.

Thiosulfate and elemental sulfur stimulated the rate of anaerobic growth of strain M4Ah and a final 2.5–3-time cell yield when cultivated with PYG under non-reduced conditions; however, only trace amounts (ca. 0.2 mM) of hydrogen sulfide were produced and the pattern of fermentation products did not change significantly. It can therefore be assumed that sulfur/thiosulfate is used by M4Ah mainly in assimilation processes. When pyruvate was used as a substrate, the growth stimulation by sulfur compounds was not detected. Sulfate, nitrate, nitrite, AQDS, arsenate, and ferrihydrite did not stimulate the growth of the isolate.

The cellular fatty acid profile of strain M4Ah was dominated by two fatty acids—C_16:0_ and C_18:0_—accounting for 35.1% and 29.1%, correspondingly (Supplementary Table S1). The other fatty acids detected (>1% of total) were C_18:1_ ω9c (6.9%), i-C_15:0_ (5.1%), ai-C_15:0_ (4.5%), 3-OH C_18:0_ (4.1%), C_22:1_ (4.0%), C_14:0_ (2.7%), C_18:1_ ω7c (2.3%), 3-OH C_16:0_ (1.8), and i-C_14:0_ (1.7%). No respiratory quinones were detected in strain M4Ah.

The strain was sensitive to streptomycin and lincomycin, but resistant to amikacin, kanamycin, neomycin, vancomycin, and ampicillin.

### 3.3. Phylogeny

**Figure 3:**
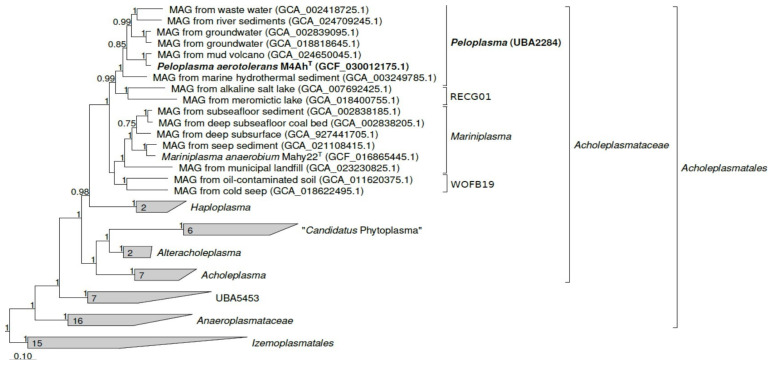
The phylogenomic placement of *Peloplasma aerotolerans* M4Ah^T^ within the family *Acholeplasmataceae* based on the phylogenetic analysis of concatenated partial amino acid sequences of 120 bacterial conservative proteins [25] by maximum likelihood inference. Bootstrap values above 90% are shown at the nodes. Bar, 0.10 changes per position.

A comparison of a full 16S rRNA gene sequence of strain M4Ah with those available in GenBank and EzBio-Cloud databases showed that the closest relative of strain M4Ah is *Mariniplasma anaerobium* Mahy22^T^ with 95.23% identity (Supplementary Figure S1). To clarify the phylogenetic position of the strain M4Ah, we performed a reconstruction based on the 120 single-copy marker bacterial proteins (bac120) [25] (Figure 3). 

According to the results obtained, the strain M4Ah belongs to the genus-level phylogenetic cluster UBA2284 (GTDB RS214) [35]. Our phylogenetic reconstruction confirms the distinctiveness of this cluster. According to GTDB RS214, the relative evolutionary divergence (RED) value for cluster UBA2284 is 0.919, which is noticeably lower than the median value of 0.925 for genus delineation. The *Mariniplasma* genus has RED of 0.930 in GTDB RS214. The RED value for a cluster that would combine strain M4Ah and *Mariniplasma anaerobium* in one genus would be 0.886, which deviates too much from the median value. 

The pairwise ANI value of the M4Ah genome and of the genome of *M. anaerobium* was 76.34% and for other closely related representatives, it ranged from 72.95 to 78.35% (Table 1), which is much lower than the threshold for prokaryotic species delineation (95%). 

We analyzed AAI values between each of the two representatives of *Mariniplasma* and UBA2284 clusters. They have rather low AAI values between each other: an average value of 61.92%, standard deviation of 0.31%, minimum value of 57.15%, and maximum value of 64.33%. Strain M4Ah had 50.46–62.81% AAI with the closest relatives; these values are below 65%—the AAI threshold proposed for the genus (Table 1). Thus, our phylogenomic analysis indicates that strain M4Ah represents a novel genus and new species within the family *Acholeplasmataceae*.

### 3.4. Genome Statistics and Analysis

The genome assembly was 2,043,143 bp in length and consisted of 88 contigs with an N50 value of 55334 bp. A total of 1935 protein-coding sequences and 41 RNA genes including 3 rRNA, 35 tRNA, and 3 ncRNA were encoded in the genome of the strain. In silico calculated G+C content of the genomic DNA was 32.4%. Genomic data on central metabolism and adaptation to oxygen were particularly analyzed.

A complete glycolysis pathway was encoded in the genome of strain M4Ah except for glucokinase, which was absent from the genome. The key enzymes of Entner–Doudoroff and pentose phosphate pathways—KDPG aldolase (MDI6452497) and glucose-6-phosphate dehydrogenase (MDI6452988, MDI6453063), respectively—were also present in the genome of strain M4Ah. The genome also contained genes encoding the biosynthesis and degradation of glycogen, one of the main carbohydrate storage pools of bacteria. The oxidative pentose phosphate pathway, providing precursors for nucleotides and amino acid biosynthesis, was complete. However, biosynthetic pathways for valine, leucine, isoleucine, threonine, alanine, asparagine, arginine, and proline were not evident, which is consistent with the inability of the strain to grow without the addition of a yeast extract. Pyridoxal-P and folate biosynthetic pathways were complete, while all other routes for vitamins and cofactors’ biosynthesis were deficient or absent from the genome of strain M4Ah.

Gluconeogenesis was most probably ineffective because one of its key enzymes—PEP-carboxykinase—was missing from the genome. The TCA cycle also did not function (only fumarate reductase was found, MDI6453383), confirming the inability of strain M4Ah to grow on tricarboxylic acids. Neither acetyl-CoA synthetase nor isocitrate lyase or malate synthase were encoded in the genome of strain M4Ah, supporting the inability of the strain to metabolize acetate. Genes, encoding acetate kinase and phosphate acetyltransferase responsible for acetate production, were found in the genome of strain M4Ah (MDI6452527 and MDI6452221, respectively). This pathway yields ATP from sugar oxidation via glycolysis and the ATP-yielding hydrolysis of acetyl-CoA to acetate via acetyl-P. The gene encoding acetaldehyde dehydrogenase was not found; however, alcohol dehydrogenase, catalyzing the production of ethanol from acetaldehyde, was encoded (MDI6452872).

The complete gene cluster for the glycine cleavage system was found in the genome of strain M4Ah (MDI6452264-67) in spite of the inability of the strain to metabolize glycine. The genome of strain M4Ah encoded several genes of aminotransferases; the subsequent degradation of the amino acid backbone proceeds through donor/ferredoxin oxidoreductases (MDI6453585, MDI6452958), yielding reduced ferredoxin as the final product. 

Acetyl-CoA could be produced under anoxic conditions by pyruvate/ferredoxin oxidoreductase (MDI6452958) during the decarboxylation of pyruvate; at the same time, genes encoding the aerobic pyruvate dehydrogenase complex were also found in the genome of the isolate.

Regarding NADH, the ubiquinone oxidoreductase respiratory complex was almost absent: only two subunits, NuoE and Nuo F (MDI6452560 and MDI6452561, respectively), were identified. Regarding complete putative sodium-translocating NADH, ferredoxin oxidoreductase (RNF complex) was found in the genome of strain M4Ah with neighboring genes organized in one cluster (MDI6452236-38, MDI6452240-41, and MDI6452243) and demonstrated high amino acid similarities (from 57.7% to 86.2%) with RnfABCDEG from *Acetobacterium woodii* [36]. Bacterial F-type ATP synthase was encoded in the strain M4Ah by a single locus (MDI6453484-91). Several hydrogenases of groups A1 and A3, typical for strict anaerobes, were found in the genome of the isolate (MD6452562 and MDI6452708, respectively).

At the same time, genes encoding [NiFe] hydrogenase of group 3, demonstrating sulfhydrogenase activity (reducing elemental sulfur with molecular hydrogen), were not found. Genes for the dissimilatory reduction of thiosulfate/sulfur (thiosulfate/polysulfide reductases of PhsA/PsrA group) were not identified. The genome of M4Ah lacks genes for the assimilatory reduction of sulfate (*cys*NC, *cys*D, *cys*H, *sir*, *cys*JI) and therefore requires a reduced source of sulfur for growth, which may reflect an adaptation to reduced anoxic environments. 

We failed to find in the genome of the strain genes encoding terminal cytochrome c oxidases of *cbb3*- and *aa3*-types as well as cytochrome *bd*-type quinol oxidase; this fact confirms the inability of the isolate to respire oxygen. The experimentally determined ability of strain M4Ah to tolerate low oxygen concentrations and the catalase activity was reflected in the genome by the presence of the corresponding catalase gene (MDI6452798). Notably, the catalase was not encoded in the genome of its closest relative, *Mariniplasma anaerobium*. Additionally, several genes coding for superoxide dismutase (MDI6453571), peroxidase (MDI6453778), and peroxiredoxins (MDI6453053, MDI6453065) were found in the genome of our isolate. 

## 4. Discussion

We have isolated a free-living, cell-wall-less, aerotolerant anaerobe—strain M4Ah—which phylogenetically belongs to the mollicutes family *Acholeplasmataceae*. Currently, this family includes genera *Acholeplasma, Alteracholeplasma*, *Haploplasma, Mariniplasma*, and *Paracholeplasma* [2]. *Acholeplasmataceae* members are mesophilic chemoorganotrophic organisms, utilizing glucose and other sugars as well as complex proteinaceous substrates as the major energy sources. All *Acholeplasmataceae* representatives are facultative anaerobes capable of microaerobic growth and fermentation, with the exception of *Mariniplasma anaerobium*, which is an obligate anaerobe (Table 2). Our data show that although strain M4Ah exhibits good growth under microaerobic conditions, it does not use molecular oxygen as an electron acceptor. The genome of strain M4Ah lacks genes encoding terminal low-affinity cytochrome c oxidases of the *caa3* type as well as high-affinity *cbb3*-type cytochrome c oxidases or *bd*-type quinol oxidases. Thus, strain M4Ah is an aerotolerant anaerobe capable of tolerating low oxygen concentrations. Aerotolerance of strain M4Ah may be caused by high activities of peroxidase, superoxide dismutase, and catalase, which can inactivate toxic products of oxygen metabolism by eventually converting them to water. Our isolate utilizes a wide range of organic substrates like simple carbohydrates and proteinaceous substrates. Moreover, a yeast extract is necessary for growth of strain M4Ah as well as for its closest relative *M. anaerobium* (Table 2). Complex growth requirements are typical for all *Acholeplasmataceae* species, showing limited anabolic abilities of acholeplasmas. All species of *Acholeplasmataceae* are mesophilic and most probably neutrophilic (literature data are scarce). It is noteworthy that neither *M. anaerobium* nor strain M4Ah are able to grow on solid media, while all other representatives of *Acholeplasmataceae* can form colonies during the growth on agar (Table 2). 

The genome of strain M4Ah is larger than that of all other members of the *Acholeplasmataceae* family and encodes the key genes of several pathways of carbohydrate degradation, ferredoxin reduction via pyruvate/ferredoxin oxidoreductase, and acetate production via acetate kinase and phosphotransacetylase. Our isolate lacks a tricarboxylic acid cycle and instead uses the anaerobic fermentation of simple sugars or proteins for substrate-level phosphorylation. Notably, the genome also contained a number of unique metabolic features including several hydrogenases and an RNF complex. The presence of hydrogenases and the electron transport complex may provide M4Ah with multiple means of cycling redox-active electron carriers like ferredoxin or NAD(P)H. The Rnf complex has been previously described from the genomes of some *Acholeplasmatales* [37]. The M4Ah genome encodes the Rnf complex of the same operon structure (rnfCDGEAB) as in *Clostridia* species [36]. As shown for fermentative bacterium *Thermotoga maritima*, during chemoorganoheterotrophic growth, the function of the Rnf complex is not primarily in energy conservation but is that of a transhydrogenase essential to balance out the electrons [38]. 

Most species of *Acholeplasmataceae* were isolated from healthy or diseased plants, insects, or vertebrates. Only in 2021 did Watanabe et al. isolate a free-living representative of *Acholeplasmataceae, Mariniplasma anaerobium,* from a brackish lake [2]. We have isolated for the first time a member of *Acholeplasmataceae* from a terrestrial mud volcano. The habitat of strain M4Ah is TMV Gnilaya Gora, located on the Taman Peninsula in southern Russia, a region with intense mud volcanic activity. Our isolate is not abundant in this TMV and constitutes approximately 0.2% of a microbial community (unpublished data). Moreover, environmental clones of the same genus as the strain M4Ah with 94.5–96.5% 16S rRNA gene similarity have been found predominantly in aquatic environments like cold seeps or lake sediments (Supplementary Table S2). The ecological function of chemoorganotrophic strain M4Ah is not clear. Although it was isolated from the culture highly enriched in “*M. alkalitolerans*”, strain M4Ah is most probably not an obligate co-partner of this methanogen in its natural habitat. There are differences in the pH optimum (7.0–7.5 for strain M4Ah and 9.0 for “*M. alkalitolerans*”), temperature optimum (30 °C for strain M4Ah and 37 °C for “*M. alkalitolerans*”) for growth, and aerotolerance, which indicates the adaptation of each strain to different environmental conditions.

**Table 2:** Characteristics of strain M4Ah and other type species of *Acholeplasmataceae* family. 1, *Peloplasma aerotolerans* (strain M4Ah, this study); 2, *Mariniplasma anaerobium* (data from [2]); 3, *Acholeplasma laidlawii* (data from [1,2,39]); 4, *Paracholeplasma morum* (data from [2,40]); 5, *Alteracholeplasma parvum* (data from [2,41]); 6, *Haploplasma axanthum* (data from [2,42]).

Characteristic	1	2	3	4	5	6
Source of isolation	Terrestrial mud volcano	Bottom water of brackish meromictic lake	Sewage, manure, humus, soil, and many animal hosts and their tissues, surfaces of some plants	Commercial fetal bovine serum and calf kidney cultures	Oral cavities and vagina of healthy horses	Murine leukemia tissue culture cells, bovine serum
Cell shapes, form, and diameter	Round, 0.32–0.65 μm	Round, 0.60–0.80 μm	Coccoid form predominates in certain cultures. Branched filaments may develop	Pleomorphic: round and coccobacillary forms, with some beaded filaments and star	Coccobacilli, 0.11–0.20 μm	Coccobacillary and coccoid
Colonies’ formation	No	No	Yes	Yes	Yes	Yes
Oxygen relationship	Anaerobic, aerotolerant	Obligately anaerobic	Facultatively anaerobic	Facultatively anaerobic	Facultatively anaerobic	Facultatively anaerobic
Temperature range, °C (optimum)	15–37 (30)	15–37 (30–32)	20–41 (37)	23–37 (35–37)	22–37 (30)	22–37 (37)
pH range (optimum)	6.5–10.0 (7.0–7.5)	6.2–8.9 (7.2–7.4)	6.5–8.5 (7.0–7.5)	ND	ND	ND
NaCl range, % (optimum)	0–4 (1)	0–5 (2–3)	ND	ND	ND	ND
Substrates utilized	Yeast extract, tryptone, pyruvate, D-glucose, D-trehalose, D-ribose, D-mannose, D-xylose, D-maltose, D-lactose, D-cellobiose, D-galactose, D-fructose, and D-sucrose. Requires yeast extract for growth. Stimulation of the growth by sulfur or thiosulfate	Yeast extract, D-glucose. Requires yeast extract for growth. Stimulation of the growth by thiosulfate	D-glucose, D-trehalose	Yeast extract, D-glucose. Requires a supplement of albumin and of palmitic acid to a serum-free medium	Yeast extract, peptone, no carbohydrates’ fermentation	D-glucose, D-maltose, D-galactose, glycogen, starch, dextrin, salicin, glycerol, D-cellobiose. Stimulation of growth with Tween 80
DNA G+C content (%)	32.42	30.10	31.7–35.7	34	29–30	28
Genome size, Mb	2.04	1.88	1.50	1.55	1.55	1.88

Likely, strain M4Ah was enriched in the co-culture with “*M. alkalitolerans*” due to the presence of ampicillin in the cultivation medium, which prevents the growth of other bacteria, and the growth of strain M4Ah is supported by the utilization of low concentrations of a yeast extract and exometabolites or necromass of “*M. alkalitolerans*”. A similar ecological role may be assigned to strain M4Ah in the microbial community inhabiting TMV. The physiological results and genome analysis indicate that M4Ah does not directly take part in the methane cycle that is dominant in TMVs. Our isolate, representing a rare part of the TMV community (0.2%), is the first species of this group, isolated in a pure culture from a mud volcano. It is a heterotroph with fermentative metabolism, utilizing simple sugars, small organic acids, and proteinaceous substrates. Strain M4Ah could consume oligomers derived from organic necromass, as it was recently shown for another organotrophic bacteria, isolated from a similar TMV [12,43]. It has also been suggested that in some TMVs, *Acholeplasmataceae* spp. may represent a component of dead insects or nematodes’ microbiomes [13]. Their predicted role in the community is likely in the fermentation of products from the degradation of organic matter to produce small molecules such as acetate or ethanol, which in turn may be utilized by other members of the TMV microbial community. The upper layers of the mud sediments in TMVs undergo mixing due to ascending gas bubbles and could meet atmospheric air. Under such conditions, the aerotolerant anaerobes like strain M4Ah and others will have adaptation advantages [12]. Strain M4Ah is the first representative of the order *Acholeplasmatales*, isolated in a pure culture from a mud volcano.

## 5. Conclusions

Therefore, based on phylogenetic position and phenotypic and genotypic characteristics of strain M4Ah, we propose to assign it to a novel species of a new genus, *Peloplasma aerotolerans* gen. nov., sp. nov.

### 5.1. Description of Peloplasma gen. nov.

*Peloplasma* (Pe.lo.plas’ma. Gr. masc. n. *pêlos*, mud; Gr. neut. n. *plasma*, anything formed or molded, image, figure; N.L. neut. n. *Peloplasma*, a mud-inhabiting form).

Gram-negative, non-spore-forming coccoid cells. Cell wall is absent. Mesophilic and neutrophilic. Anaerobic, chemoorganotrophic. The type species is *Peloplasma aerotolerans*. 

### 5.2. Description of Peloplasma aerotolerans sp. nov.

*Peloplasma aerotolerans* (ae.ro.to’le.rans. Gr. masc. n. *aêr*, air; L. pres. part. *tolerans*, tolerating; N.L. part. adj. *aerotolerans*, tolerating air).

Cells are non-motile coccoid with the diameter of about 0.32–0.65 μm and filterable through 450 nm membranes. Aerotolerant anaerobe. Grows at 15–37 °C (optimum: 30 °C), at pH 6.5–10.0 (optimum: 7.0–7.5) and at NaCl concentrations of 0–40 g L^−1^ (optimum: 10 g L^−1^). A yeast extract is necessary for growth. Growth is enhanced by elemental sulfur or thiosulfate. Grows by the fermentation of the yeast extract, tryptone, pyruvate, D-glucose, D-trehalose, D-ribose, D-mannose, D-xylose, D-maltose, D-lactose, D-cellobiose, D-galactose, D-fructose, and D-sucrose. No growth was observed on peptone, glycine, glutamate, citrate, succinate, fumarate, malate, lactate, casamino acids, beef extract, glycerol, acetate (microaerobically), D-raffinose, formate, L-arabinose, i-inositol, propionate, crotonate, and CO_2_:H_2_ under anaerobic conditions. Catalase-positive, microaerobic conditions are preferable for the strain. The major cellular fatty acids are C_16:0_ and C_18:0_. The genome of the type strain is characterized by the size of 2.04 Mb and a G+C content of 32.4%. The type strain, M4Ah^T^ (=DSM 112561^T^ = VKM B-3485^T^ = UQM 41475^T^), was isolated from a terrestrial mud volcano, Taman Peninsula, Russia. The GenBank accession number for the 16S rRNA gene sequence of strain M4Ah^T^ is OR436924. This Whole Genome Shotgun project has been deposited in DDBJ/ENA/GenBank under the accession JASCXW000000000.

## Figures and Tables

**Table 1:** Pairwise ANI and AAI values between strain M4Ah and closest relatives of *Acholeplasmataceae* [32,33]. Genome of strain M4Ah Genbank accession number is ASM3001217v1.

Microorganism	GenBank Accession No.	ANI, %	AAI, %
*Acholeplasma laidlawii* DSM 23060	ASM338576v1 **	75.09	50.46
*Acholeplasma equifetale* ATCC 29724	ASM68773v1	72.95	51.18
*Acholeplasma hippikon* ATCC 29725	ASM70276v1	74.64	51.58
*Acholeplasma equirhinis* N93	ASM1705265v1	73.78	51.31
*Mariniplasma anaerobium* Mahy22^T^	ASM1686544v1 **	76.34	62.81
*Paracholeplasma morum* ATCC 33211	ASM1690705v1 **	75.31	52.34
*Paracholeplasma manati* Oakley	ASM2574299v1	77.40	52.66
*Paracholeplasma vituli* 92-19	ASM2544693v1	78.35	52.11
“*Alteracholeplasma palmae*” J233	ASM96805v1 *	75.31	52.78
*Haploplasma axanthum* NCTC10138	LR215048.1 **	75.57	51.77
*Haploplasma modicum* ATCC 29102	ASM68783v1	74.55	51.93

* Genomes of the closest relatives *Alteracholeplasma parvum* and *Haploplasma cavigenitalium* are not available. ** Genomes of the type species of the family *Acholeplasmataceae*.

## Data Availability

The GenBank accession number for the 16S rRNA gene sequence of strain M4Ah^T^ is OR436924. This Whole Genome Shotgun project has been deposited in DDBJ/ENA/GenBank under the accession JASCXW000000000.

## Supplementary Materials

The following supporting information can be downloaded at: https://www.mdpi.com/article/10.3390/life14050563/s1. Figure S1: Phylogenetic placement of *Peloplasma aerotolerans* M4Ah^T^ based on 16S rRNA gene sequence; Table S1: The cellular fatty acid profile of M4Ah^T^ strain; Table S2: Distribution of bacterial clones species-related to M4Ah^T^ isolate in environmental and artificial habitats. References [28,30,44,45,46,47,48,49,50,51,52,53].
