# Supplementary material for: Peloplasma aerotolerans gen. nov., sp. nov., a Novel Anaerobic Free-Living Mollicute Isolated from a Terrestrial Mud Volcano
Source: Life (Basel). 2024 Apr 26;14(5):563. doi: 10.3390/life14050563 (PMC11122141; doi:10.3390/life14050563)
Supplement: Supplementary file 1 [file life-14-00563-s001.zip › life-2960354-supplementary.pdf]

Peloplasma aerotolerans gen. nov., sp. nov., a novel anaerobic free-living mollicute isolated from a terrestrial mud volcano

Maria A. Khomyakova[1*], Alexander Y. Merkel[1], Andrei A. Novikov[2], Alexander I. Slobodkin[1]

[1]Winogradsky Institute of Microbiology, Research Center of Biotechnology of the Russian Academy of Sciences, Leninskiy Prospect, 33, bld. 2, 119071, Moscow, Russia

[2]Gubkin University, Leninskiy Prospect, 65/1, 119991, Moscow, Russia

[*]Author for correspondence: Maria Khomyakova. Tel: +7 499 135 8101.

e-mail: mary_klimova@mail.ru

Supplementary Table S1. The cellular fatty acid profile of M4Ah[T] strain.

Name	ECL (HP-5MS)	Strain M4Ah
i-$C_{14:0}$	13.647	1.7
$C_{14:0}$	14.000	2.7
i-$C_{15:0}$	14.635	5.1
ai-$C_{15:0}$	14.692	4.5
i-$C_{16:0}$	15.632	1.9
$C_{16:0}$	16.000	35.1
ai-$C_{17:0}$	16.718	ND
$C_{17:0}$	17.000	0.8
3-OH $C_{16:0}$	17.508	1.8
$C_{18:1}$ ω9c	17.762	6.9
$C_{18:1}$ ω7c	17.816	2.3
$C_{18:0}$	18.000	29.1
3-OH $C_{18:0}$	19.538	4.1
$C_{22:1}$	21.778	4.0

Supplementary Table S2. Distribution of bacterial clones species-related to M4Ah^T isolate in environmental and artificial habitats. Blastn with the identity>94.5% and alignment length ≥1 300 bp, was used in order to align the metagenomic quality-filtered shotgun reads against strain M4Ah^T genome.

Database name	GenBank accession number	Source	16S rRNA sequence similarity, %	Reference
Stn1_Nov_17	KX014197.1	Surface water of mangrove creek, India	96.26	44
EMIRGE_OTU_s3t2d_423	JX222651.1	Subsurface aquifer sediment, USA	96.12	Unpublished
EV818CFSSAHH219	DQ337019.1	Subsurface water, South Africa	95.99	45
B98	AB874577.1	Anaerobic reactor treating corn straw, China	96.47	46
FGL12_B14	FJ437874.1	Lake sediment, USA	95.58	47
MZ-XQ	CP017950.1	Deep subseafloor coal bed, Japan	95.58	Unpublished
Ach1	MW599757.1	Cold seep sediment, China	95.51	Unpublished
TANB25	AY667255.1	TCE-dechlorinating groundwater, USA	95.77	48
zrk7	OP108612.1	Cold seep, China	95.24	Unpublished
XX0065	FJ820457.1	Hypertrophic freshwater lake, China	95.04	Unpublished
CK06-06_Mud_MAS1B-28	AB369171.1	Riser drilling mud fluid, Japan	95.50	49
e871f63d-8a2d-4cdd-b754-05539c673417*	OY764060.1	Ciliate metagenome, UK	94.76	Unpublished

SAW1_B33	FJ716289.1	Water column, at 10.3 m water depth, USA	94.64	Unpublished
B-6	HQ703858.1	Lake sediment, China	95.01	50
HJ1A286	KC852988.1	Soda lake sediment, Mongolia	96.18	51
BF65B_B15	HM141299.1	Supraglacial spring outflow, USA	96.54	52
2P9SHNG554	FJ469288.1	Oil well, USA	95.95	53

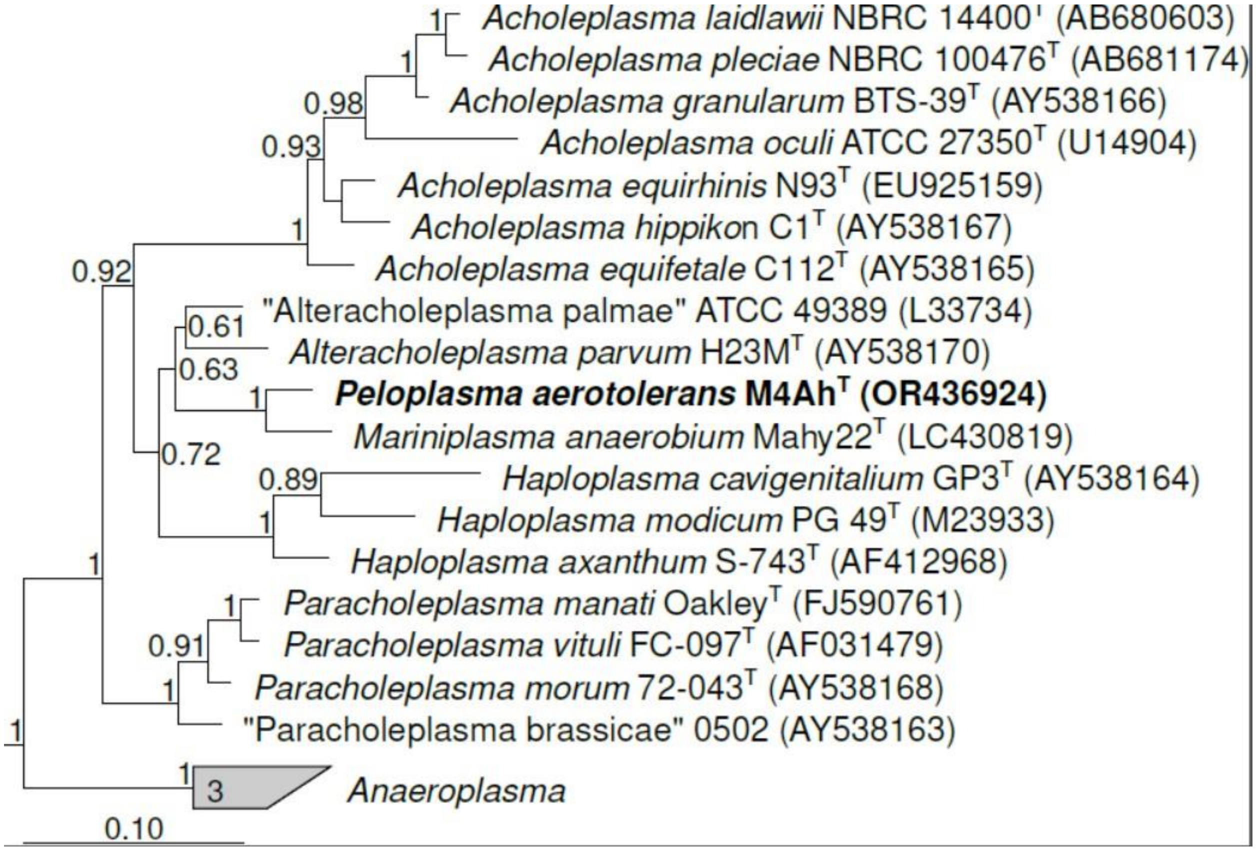

Supplementary Figure 1. Phylogenetic placement of *Peloplasma aerotolerans* M4Ah^T based on 16S rRNA gene sequence. The tree was built using the IQ-TREE program [28] and the ultrafast approximation for phylogenetic bootstrap [30]. Bootstrap values above 70% are shown at the nodes. Bar, 0.10 changes per position.
